# Reexpansion pulmonary edema following a posttraumatic pneumothorax: a case report and review of the literature

**DOI:** 10.1186/1749-7922-6-32

**Published:** 2011-09-02

**Authors:** Mark Malota, Markus C Kowarik, Barbara Bechtold, Reinhard Kopp

**Affiliations:** 1 Krankenhaus München Harlaching, Departement of Surgery, Munich, Germany; 2 Klinikum Rechts der Isar, Munich, Germany; 3 Krankenhaus München Harlaching, Departement of Anaesthesiology, Munich, Germany

**Keywords:** reexpansion pulmonary edema, acute pulmonary failure, posttraumatic pneumothorax, chest tube

## Abstract

The reexpansion pulmonary edema is a rare, but life threatening complication of a pneumothorax. Early recognition and a fast symptom orientated therapy are necessary for a good outcome. Several cases after non traumatic pneumothoraces are reported. We describe a patient who presented with a post-traumatic right pneumothorax. After the insertion of a chest tube he developed a reexpansion pulmonary edema, which had to be treated by an intubation.

Additionally, a review of the literature regarding case reports of reexpansion pulmonary edema is presented.

## Background

Cases of posttraumatic or spontaneous pneumothorax are usually treated by the insertion of a chest tube. A rare, potentially life-threatening complication of pneumothorax drainage is the pulmonary reexpansion edema. Usually it occurs after non traumatic pneumothoraces. Early recognition and a fast symptom orientated therapy of pulmonary reexpansion edema are necessary for a good outcome. Here we present a case of the development of a reexpansion pulmonary edema after a traumatic pneumothorax

## Case Presentation

A 21-year-old male, sportive patient was admitted to our surgical emergency department after he had been involved in a traffic accident. As the unbelted driver of a car, he crashed frontally against another car with approximately 50 km/h.

On first sight he was complaining of jabbing pain in the right hemothorax and in the sternal region, thoracic constriction and a considerable dyspnoea. Apart from that, he had signs of a beginning cold: since two days he had a cough and suffered from an acute rhinitis. The patient was an occasional smoker but did not have any history of pulmonary or other diseases.

The asthenic man (weight 62 kg, size 179 cm) was orientated and had no neurological deficit with stable vital parameters. Some small superficial wounds and haematoma in the lower part of the sternum and the right hemithorax could be found. Furthermore a discrete cyanosis of the lips was seen. On auscultation, the patient was found to have no respiratory murmur and hyperresonant percussion on the right side, with the left lung completely normal.

**Figure 1:**
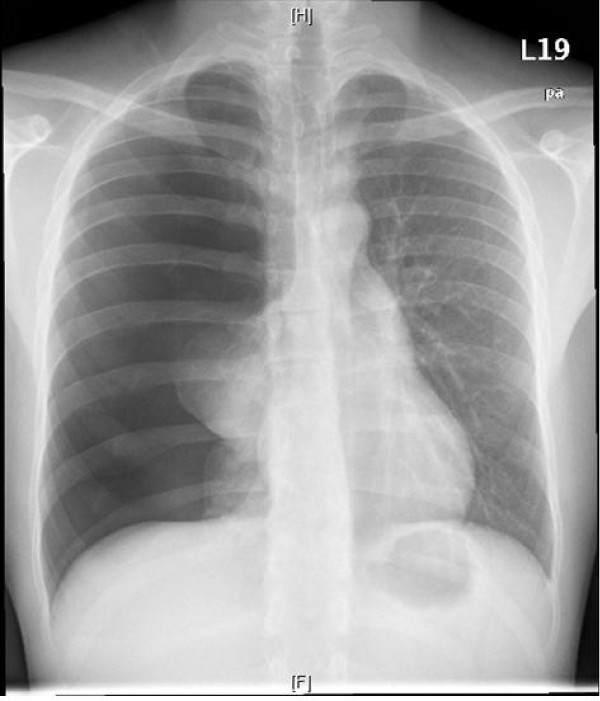
**The first chest x-ray**. We find a pneumothorax on the right with a subtotal lung collapse.

Using a chest x-ray, we saw a pneumothorax on the right with a subtotal lung collapse (Figure 1).

Under insufflation of 4 l O1/2/min, the arterial blood gas showed signs of a respiratory partial insufficiency: the pO^2 ^was 50 mmHg and pCO^2 ^43 mmHg. Apart from a leucocytosis of 17, 9 mg/dl, the blood examination was without pathological findings.

Based on the diagnosis of a posttraumatic pneumothorax we immediately performed the insertion of a chest tube in Buelau technique located in the 5^th ^ICR, proximal axillary line under local anaesthesia, and connected it to a 3-chamber chest drain system with suction of 20 cm water column. The pre-treatment time took approximately twenty minutes.

The pulmonary condition of the patient ameliorated (pO^2 ^72 mmHg, pCO^2 ^38 mmHg), both lungs were ventilated and SpO1/2 increased ten minutes after the intervention up to 99%. Because of a moderate analgesic and sedative medication, we kept the patient for further monitoring in our anaesthetic recovery room. Here the patient reported only light pain at the entrance of the drainage, without having any dyspnoea.

Two hours later, the patient's condition rapidly worsened. He was pale, sweating, tachypnoic and complained of increasing chest pain with dyspnoea. In spite of 10 l/min O1/2, the SpO1/2 was only 82% with a heart rate of 122/min and a decreasing blood pressure. Checking the arterial blood gas, the pO^2 ^was 61 mmHg and pCO^2 ^58 mmHg, indicating now a global respiratory failure

**Figure 2:**
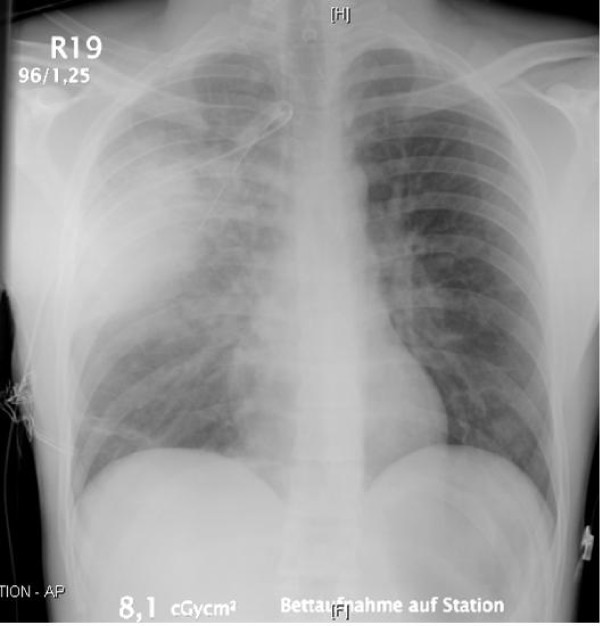
**The second chest x-ray with the thoracic drain**. The lung is correctly expanded. There is a suspect shadow along the lateral right chest wall.

Immediately a chest x-ray was taken (Figure 2). Although the lung seemed expanded correctly, there was a suspect shadow along the chest wall, where the tube was entering. Because of the suspicion of a haematoma of the thoracic wall, we checked the haemoglobin, which was stable at 14 g/dl. Furthermore there was no blood in the tube. Meanwhile the patient's condition got worse progressively, so that we decided to initiate an intubation to be able to improve the oxygenation using mechanical respiration. At the inspection of the pharynx, an immense amount of suppuration was blocking the upper respiratory tract. Finally 350 ml of putrid mucos were sucked off, whereupon a tracheal intubation could be performed. Now the mechanical ventilation of the patient was easy to handle and in the following twenty minutes another 300 ml mucos were removed.

**Figure 3:**
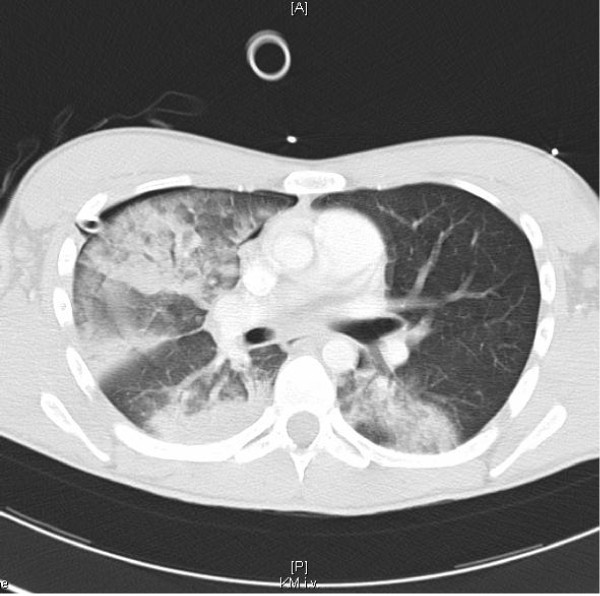
**CT findings of the lung edema**. A bilateral lung edema can be seen in the CT of the chest.

After that, we did a CT scan of the thorax, which surprisingly showed a marked ipsilateral lung edema, designated as a reexpansion pulmonary edema. Furthermore a small edema was seen on the contralateral side, however without any other pathological findings concerning the lung or the complete rib cage (Figure 3).

The patient was rapidly stabilized under automatic continuous positive airway pressure respiration (CPAP) and short-term therapy with Noradrenaline and Furosemid. After transferring the patient to our intensive care unit, the respiratory and haemodynamic situation remained stable.

Under a calculated antimicrobiotic therapy with Piperacilin and Sulbactam the respiratory condition quickly improved and the patient could be extubated after 48 hours. Chest tubes could be removed soon and the patient was released from hospital on the 4^th ^post OP day with normally expanded lung.

## Discussion

"Reexpansion pulmonary edema" (RPE) has been described as a rare, life threatening complication in the treatment of lung atelectasis, pleural effusions or spontaneous pneumothorax with a mortality up to 20% [1].

Pinault in Paris was the first to describe the clinical situation in 1853 after the drainage of 3 l pleural effusion [2]. The first report of a RPE after treatment for a totally collapsed lung because of pneumothorax was published in 1958 by Carlson [3]. In the following years, there were several cases reporting on the occurrence of RPE after spontaneous pneumothorax, the resection of a mediastinal tumor, thoracoscopy, or talc pleurodesis [3-5].

Mahfood et al reviewed all reported cases from 1958 to 1987 with 47 cases of RPE. Here the clinical disorders occur from almost free of complaints to foydurant processes with lethal ending. A rapid onset of dyspnoea is the cardinal symptom, followed by cough and hypotension. Risk factors seem to be age (the younger the patient, the higher the risk), female sex, degree of lung collapse, a pneumothorax existing more than 24 hours, a reexpansion of the lung in less than ten minutes, using a suction system and - in cases of a pleural effusion - an evacuation volume of more than 2000 ml [1].

RPE can occur as well after talc pleurodesis. In a retrospective study of 614 patients, 12 patients developed transient interstitial opacities on the chest x-ray, indicating a RPE [4].

In one case report, RPE occurred after left thoracoscopic resection of a mediastinal tumor. Here, the lung had been preoperatively compressed by the tumor and one-lung ventilation was used [5].

Fujino et al reported an intraoperative RPE during a video assisted thoracoscopy, where high-frequency jet ventilation was used to reexpand the lung, which had collapsed 23 days before [6]. All cases had in common that the duration of the lung collapse was at least 12 hours.

Although the precise incidence of RPE is not known, it is generally considered to be very low. A series of 320 cases of spontaneous pneumothorax was published by Rozenman et al in 1996 with 3 cases of RPE [7]. In another case series of a combined total of 775 cases with patients treated for spontaneous pneumothorax was no reported case of RPE [8,9].

Usually, most of the reported cases were described as an ipsilateral RPE, but there are few patients with contra- or even bilateral edema, which seem to raise the mortality. Her and Mandy published 3 cases with a contralateral RPE after a right upper lobectomy for cancer, a drainage of a pleural effusion and an intraoperative collapse during non thoracic surgery. In all cases, the RPE in the contralateral lung occurred faster and more severe than in the collapsed side [10].

Appraising the temporal dynamic, the first symptoms often occur within the first hour up to 24 hours after the re-expansion of the lung [7].

As we know from a 22 case series published by Gleeson, who reviewed the CT scans of patients with RPE, the most common CT findings of reexpansion pulmonary edema include ipsilateral ground-glass opacities, septal thickening, foci of consolidation and areas of atelectasis [11].

The aetiology depends on multiple factors; however the pathophysiological process has not yet been completely explored. From several animal experiments it could be seen, that a chronic lung collapse causes a thickening of the capillary endothelium by the release of MCP1 (monocyte chemoattractant protein 1), Leukotriene B4 and IL-8 (Interleukin 8). On reexpansion of the lung, the microvessels are suddenly stretched, which harms their endothelium. Thereby the capillary permeability is increased and a loss of alveolar surfactant can be observed. Thus the perivascular pressure of the microvessels decreases, which leads to further endothelial damage. In addition to that, it could be demonstrated, that oxidases are induced, which leads to apoptosis of alveolar and endothelial cells [12,13].

The treatment for RPE is symptomatic. Apart from monitoring the patient's vital parameters, invasive respiration with a high positive end-expiratory pressure may be necessary to reexpand the collapsed alveoli. Supportively anti-inflammatory drugs and diuretics should be given [14].

## Conclusion

Although the RPE is a rare complication after the treatment of a pneumothorax, the physician should be aware of the severity of this disease pattern and always keep it in mind. Furthermore he should be aware of the fact that it can as well occur after a traumatic pneumothorax.

## Consent

Written informed consent was obtained from the patient for publication of this Case report and any accompanying images. A copy of the written consent is available for review by the Editor-in-Chief of this journal.

## Competing interests

The authors declare that they have no competing interests.

## Authors' contributions

MM drafted the manuscript. MCK made substantial revisions. BB searched the literature and the findings. RK had given final approval of the version to be published. All authors read and approved the final manuscript.
